# Deep sequencing of multiple regions of glial tumors reveals spatial heterogeneity for mutations in clinically relevant genes

**DOI:** 10.1186/s13059-014-0530-z

**Published:** 2014-12-03

**Authors:** Akash Kumar, Evan A Boyle, Mari Tokita, Andrei M Mikheev, Michelle C Sanger, Emily Girard, John R Silber, Luis F Gonzalez-Cuyar, Joseph B Hiatt, Andrew Adey, Choli Lee, Jacob O Kitzman, Donald E Born, Daniel L Silbergeld, James M Olson, Robert C Rostomily, Jay Shendure

**Affiliations:** Department of Genome Sciences, University of Washington, Seattle, WA 98195 USA; Division of Medical Genetics, University of Washington, Seattle, WA 98195 USA; Department of Neurosurgery, University of Washington, Seattle, WA 98195 USA; Clinical Research Division, Fred Hutchinson Cancer Research Center, Seattle, WA 98109 USA; Department of Pathology, University of Washington, Seattle, WA 98195 USA

## Abstract

**Background:**

The extent of intratumoral mutational heterogeneity remains unclear in gliomas, the most common primary brain tumors, especially with respect to point mutation. To address this, we applied single molecule molecular inversion probes targeting 33 cancer genes to assay both point mutations and gene amplifications within spatially distinct regions of 14 glial tumors.

**Results:**

We find evidence of regional mutational heterogeneity in multiple tumors, including mutations in *TP53* and *RB1* in an anaplastic oligodendroglioma and amplifications in *PDGFRA* and *KIT* in two glioblastomas (GBMs). Immunohistochemistry confirms heterogeneity of *TP53* mutation and *PDGFRA* amplification. In all, 3 out of 14 glial tumors surveyed have evidence for heterogeneity for clinically relevant mutations.

**Conclusions:**

Our results underscore the need to sample multiple regions in GBM and other glial tumors when devising personalized treatments based on genomic information, and furthermore demonstrate the importance of measuring both point mutation and copy number alteration while investigating genetic heterogeneity within cancer samples.

**Electronic supplementary material:**

The online version of this article (doi:10.1186/s13059-014-0530-z) contains supplementary material, which is available to authorized users.

## Background

Regional heterogeneity of mutations has been observed in a variety of tumor types [1,2]. This intratumoral heterogeneity has broad implications for the clinical management of cancer patients, especially in the current paradigm of personalized medicine based on genomic analysis of a single cancer biopsy. Within the context of primary brain tumors, several groups have previously identified heterogeneity of gene amplifications in genes *EGFR* and *PDGFRA* in glioblastoma multiforme (GBM) using fluorescence *in situ* hybridization (FISH) and array-comparative genomic hybridization on multiple regions within primary tumors [3,4]. Despite the dropping cost of DNA sequencing, however, the extent of point mutational heterogeneity in brain tumors remains limited to a single case of GBM [5]. This is in part because the investigation of intratumoral heterogeneity requires both sampling and deep sequencing of multiple regions in a tumor.

We recently developed a method to identify low frequency mutations across known cancer genes [6] using the single molecule molecular inversion probe (smMIP) assay, which combines multiplex target capture with single molecule tagging [6,7]. Here, we extend this technique to detect gene amplifications and examine intratumoral heterogeneity by targeting 33 cancer genes across 62 spatial sections of 14 glial tumors, including 10 grade IV gliomas (all GBMs), three grade III gliomas (one each of ependymoma, astrocytoma, and anaplastic oligodendroglioma) and one grade II astrocytoma. We detected intratumoral heterogeneity in both point mutations and amplifications of genes implicated as glioma tumor drivers and therapeutic targets.

## Results

### Study design

**Figure 1:**
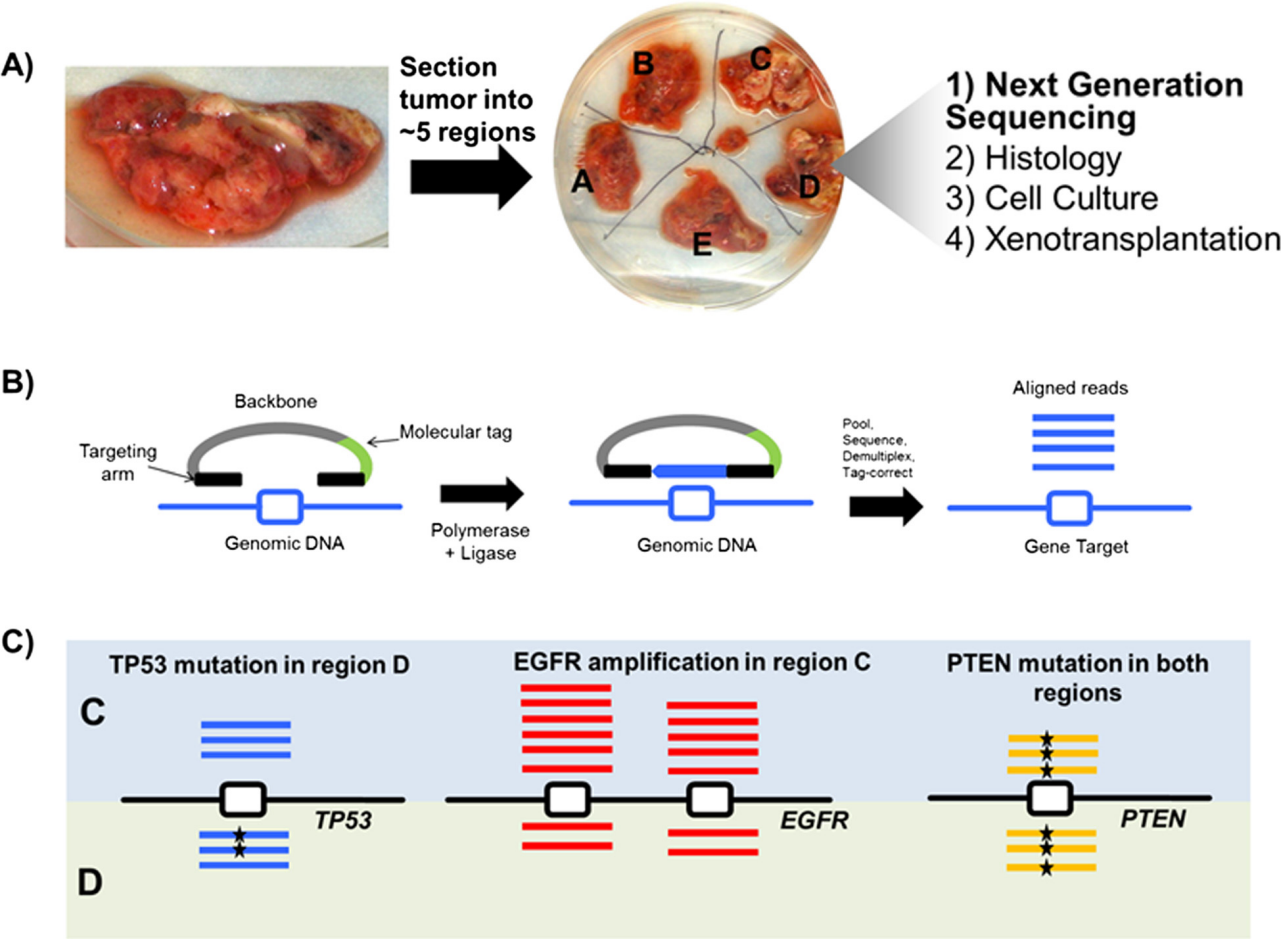
**Experimental approach. (A)** Each tumor was divided into three to five regions to assay intratumoral heterogeneity. Each individual region was subdivided into four pieces for use in next generation sequencing (NGS), histology, cell culture and xenotransplantation. **(B)** Molecular inversion probe method. Oligonucleotide probes were previously designed against 33 cancer genes [6]. MIPs have a common backbone sequence, molecular tag sequence as well as targeting arms homologous to regions flanking targets of interest. After polymerase extension and ligation, targeted sequence is captured within a circular molecule. Captured sequences are amplified in a barcoding-PCR reaction and multiple samples are pooled and sequenced on the same lane. After tag-correction (not shown), reads corresponding to each tumor region are mapped to the human reference sequence to be used to identify copy number amplifications and point mutations specific to one region or another. Additional details are provided in Figure S1 in Additional file 1. **(C)** Example of comparisons: MIP captures of regions C and D can detect both *TP53* point mutation heterogeneity and *EGFR* amplification heterogeneity within a tumor. Tumors with mutational heterogeneity were required to share either a point mutation or copy number alteration (in this case mutation of *PTEN*) across all regions to ensure that differences in observed mutation were not due to varying levels of tumor cellularity.

To assess heterogeneity within gliomas, we dissected each of 14 tumors into 3 to 5 regions per tumor (Figure 1A; Table S1 in Additional file 1). We used the smMIP assay on genomic DNA isolated from each region to identify single nucleotide variants and high level copy amplifications (Figure 1B; Figure S1 in Additional file 1). smMIP probes capture target sequence into covalently linked circular molecules after polymerase extension and ligation. Following barcoding-PCR, sample pooling, sequencing, deduplication and alignment, we identified high level amplifications and point mutations (Figure 1B,C; Figure S1 in Additional file 1).

**Figure 2:**
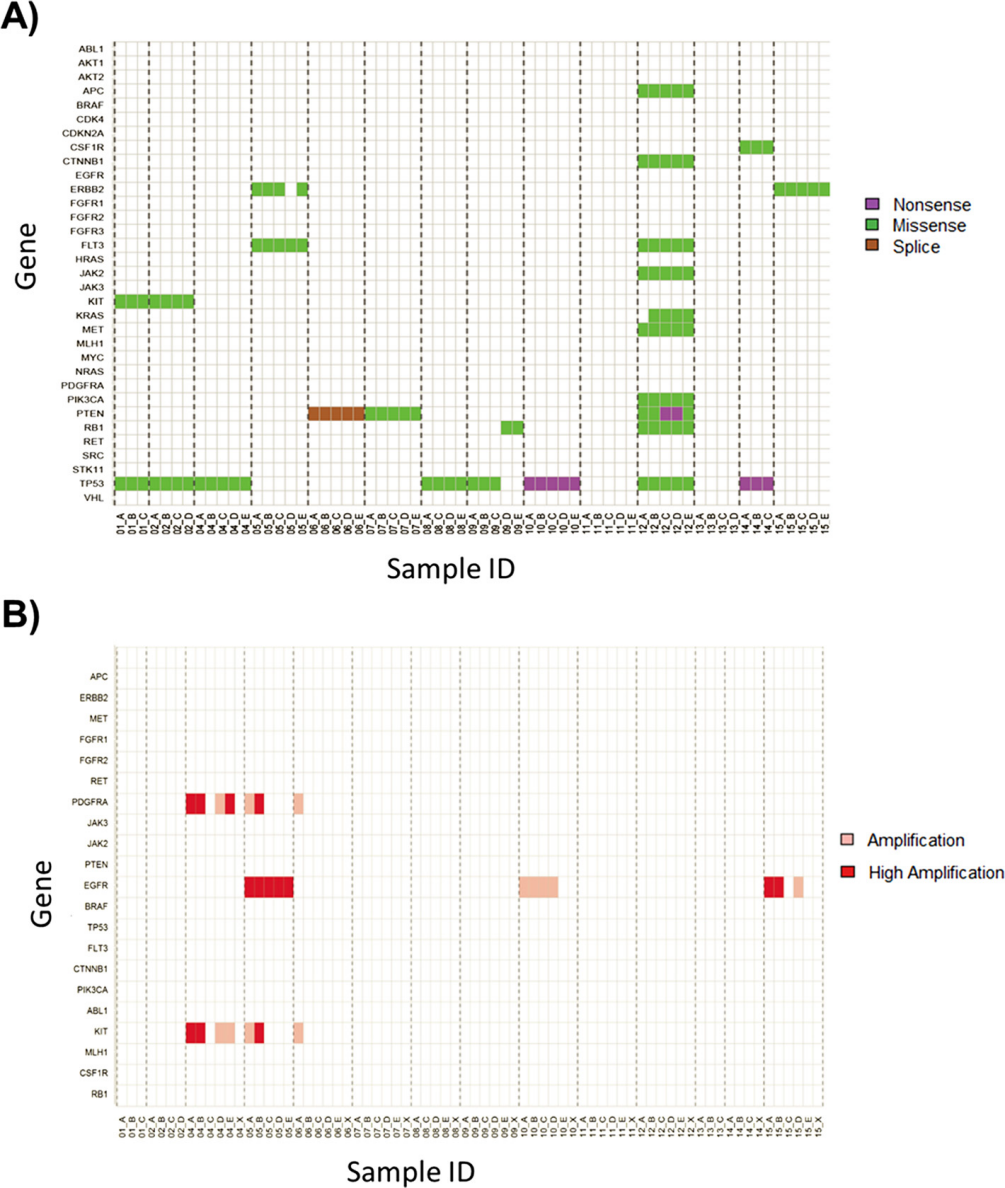
**Summary of heterogeneity observed across all samples. (A)** Protein-altering mutations detected across all tumor regions. Genes mutated twice in the same tumor region are not identified here but can be found within a table of all mutations (Table S3 in Additional file 1). **(B)** High level gene amplifications detected by smMIP assay. Copy number was estimated by comparing all tumor samples against 12_X, a universal control from BI12 (see Figure S2 in Additional file 1 for analysis using patient matched controls). ‘Amplification’ indicates genes with coverage three-fold higher than median coverage across a sample. ‘High Amplification’ indicates genes with coverage six-fold higher than median coverage across a sample. Region X refers to brain tissue grossly uninvolved by tumor. Our approach would miss any low-level gene amplifications within these tumors.

Across the 14 tumors and 33 genes considered in this analysis, we identified a total of 33 putative protein-altering mutations (Tables S1 and S2 in Additional file 1). Tumors had between zero and 16 putative protein-altering mutations, with a median of two. *TP53* was the most commonly mutated gene, with mutations found in 8 out of 14 tumors (Figure 2A; Table S3 in Additional file 1). One tumor, BI12, had many more candidate somatic mutations than other tumors (n = 16 versus median n = 2 in other tumors). Mutations in this GBM were predominantly G > T (or C > A) transversions (8 of 16 total), possibly representing mutation from unrepaired 8-oxo-guanine damage. Most mutations were observed across all tumor regions of BI12, consistent with a defect in DNA repair arising early in the development of the tumor.

To identify high level gene amplifications in tumors, we compared read depth of smMIP-targeted regions in each tumor against that of a control tissue. As smMIP sequencing suggested that a subset of control tissues were contaminated with tumor cells, we performed analyses using either patient-matched controls (Figure S2 in Additional file 1) or a ‘universal’ control (Figure 2B). For the latter we selected control tissue from tumor BI12, as it appeared to have the least tumor contamination based on allele fraction of known pathogenic point mutations, and restricted copy number analyses to targets with >30× coverage in control tissue from BI12 as well as targets whose GC percentage ranged from 30 to 60% (n = 885 capture probes). A careful review of discrepant calls when using patient-matched versus a universal control indicated that use of the universal control was more sensitive in identifying *bona fide* amplification events (as confirmed with Taqman assays) secondary to the contamination of a subset of control tissues with tumor cells. After applying our filters (see Materials and methods), a total of 21 genes could be assayed in a total of 62 regions across 14 tumors (Figure 2B).

The ratio of coverage of each probe was calculated relative to the control tissue (from BI12). We used DNACopy [8] to segment genes and obtain R, the mean ratio of coverage relative to control for each gene. We estimated the copy number for each gene by dividing R for each gene by the median value of R across all genes for each tissue. Genes with ratios above 3 were called as amplified. Genes with ratios above 6 were called as highly amplified. We did not measure deletion of genes using this method.

This process identified five tumors with gene amplifications, with three having one or more regions with a highly amplified gene (Figure 2B). Three tumors had amplification of both *PDGFRA* and *KIT*, and three tumors had amplification of *EGFR*. We validated copy number estimates for a subset of calls using a variety of different methods, including Taqman quantitative PCR (qPCR; across all tumors for *EGFR* and tumors BI05, BI06 and BI15 for *PDGFRA*), as well as whole genome sequencing (in tumor BI15 for *EGFR*). MIP copy number estimates of *EGFR* were highly correlated (R^2^ = 0.90) with delta Ct obtained by Taqman qPCR when compared across all 62 regions sequenced (Figure S3 in Additional file 1). Additionally for five tumor regions of BI15 that were subjected to light-whole genome sequencing, *EGFR* copy number estimates were consistent between whole genome sequencing and smMIP techniques (Supplementary methods and Figure S4 in Additional file 1).

Tumors in which only a subset of regions possess an amplification or point mutation with no other mutation shared across regions can be the result of either mutational heterogeneity within a tumor or varying levels of tumor content between different tumor regions. As an example, tumor BI15 was called as amplified for *EGFR* in two out of five regions with no other somatic mutations/point mutations detected across the tumor (Figures S5 and S6 in Additional file 1). Upon close inspection of histologic slides prepared from adjacent tissue, the observed difference in amplification was most likely due to lower tumor cellularity within other regions of this tumor rather than intratumoral genetic heterogeneity. This was also seen in tumor BI04, where one region without detectable *PDGFRA* amplification also had lower frequencies of a *TP53* mutation seen across all regions. For this reason, we chose to restrict our interpretation of intratumoral heterogeneity to tumors in which all regions also shared a point mutation or gene amplification. Three tumors met these criteria and are described below.

### Spatial heterogeneity of *TP53* and *RB1* point mutations

**Figure 3:**
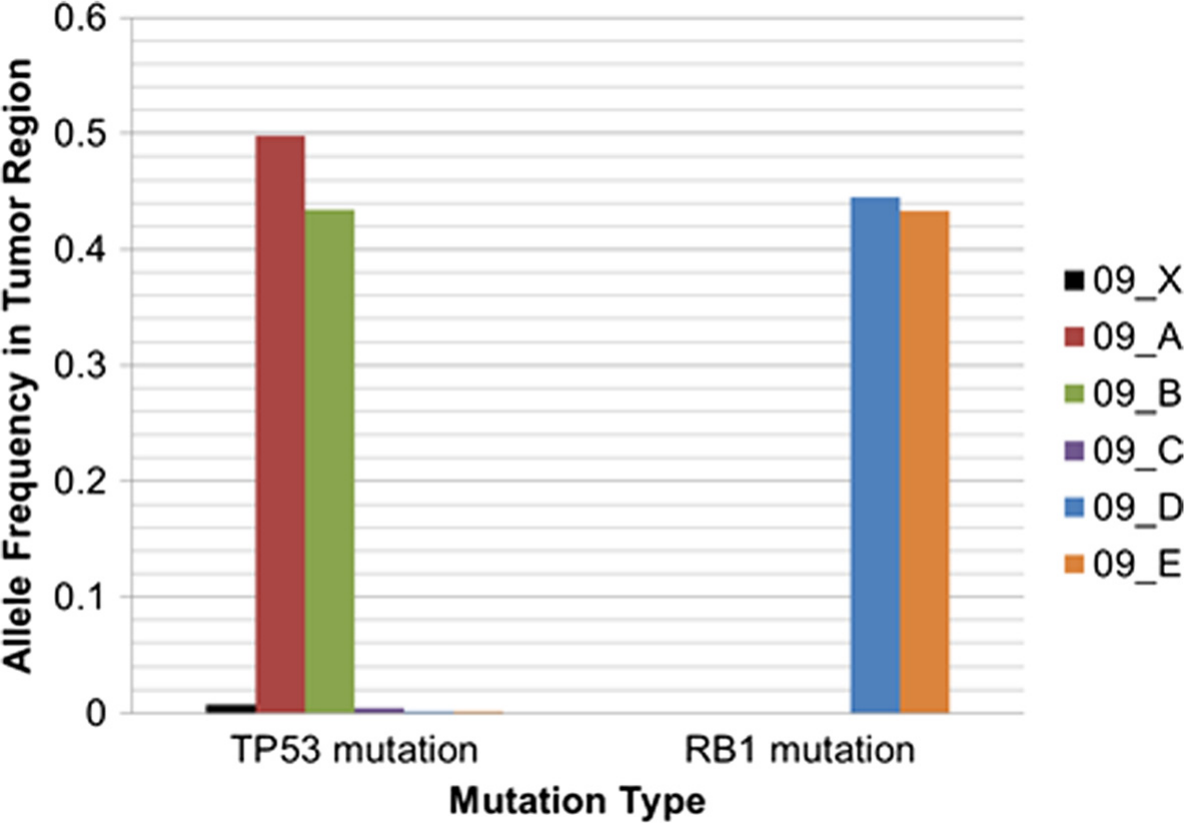
**Intratumoral heterogeneity of** ***TP53*** **and** ***RB1*** **determined from smMIP sequencing.** Tumor BI09 was sectioned into five regions (A to E). Brain tissue grossly uninvolved by tumor was used as a control (X). Each region was assayed for mutations in 33 genes, including *TP53* and *RB1*. This plot shows the allele balance of *TP53* and *RB1* mutations within each tumor region. Regions A and B have a high allele fraction mutation in *TP53*, while regions D and E have a high allele fraction mutation in *RB1*. Sanger results validated *TP53* and *RB1* mutations in each region and also revealed that all regions shared a R132H mutation in *IDH1* (Figure S7 in Additional file 1).

One tumor exhibited clear spatial heterogeneity with respect to point mutations within the 33 genes investigated (Figure 3). BI09, an *IDH1*-mutant anaplastic oligodendroglioma, had a high allele fraction (>30% reads supporting mutation) inactivating mutation (R248H) in *TP53* in only two regions of the tumor (A and B). This tumor had high allele fraction mutations in *RB1* exclusively in two other regions (D and E) within the same tumor. Both *TP53* and *RB1* mutations were present at trace levels (<1%) within region C. As clinical workup indicated that BI09 had an *IDH1* mutation, we investigated all regions of this tumor by Sanger sequencing and found that regions A to E shared the *IDH1* R132H mutation. Sanger sequencing also validated the *TP53* mutation in regions A and B as well as the *RB1* mutation in regions D and E (Figure S7 in Additional file 1). Immunohistochemistry of p53 and IDH1-R132H expression on tissue adjacent to regions A to E provided additional confirmatory evidence (Figure S8 in Additional file 1). These findings are consistent with an *IDH1*-mutant tumor subsequently diverging to form subclones with mutations in *RB1* and *TP53* [9,10]. A neuropathologist (LFG-C) scored the grade and diagnosis for each of these samples blinded to the mutation type. Interestingly, the presence of *TP53* mutation correlated with the higher grade histology (Table S4 in Additional file 1). The clinical significance is unknown but this serves as a potential example of how genomic heterogeneity may affect histology of a tumor.

### Spatial heterogeneity of *PDGFRA* and *KIT* amplifications

**Figure 4:**
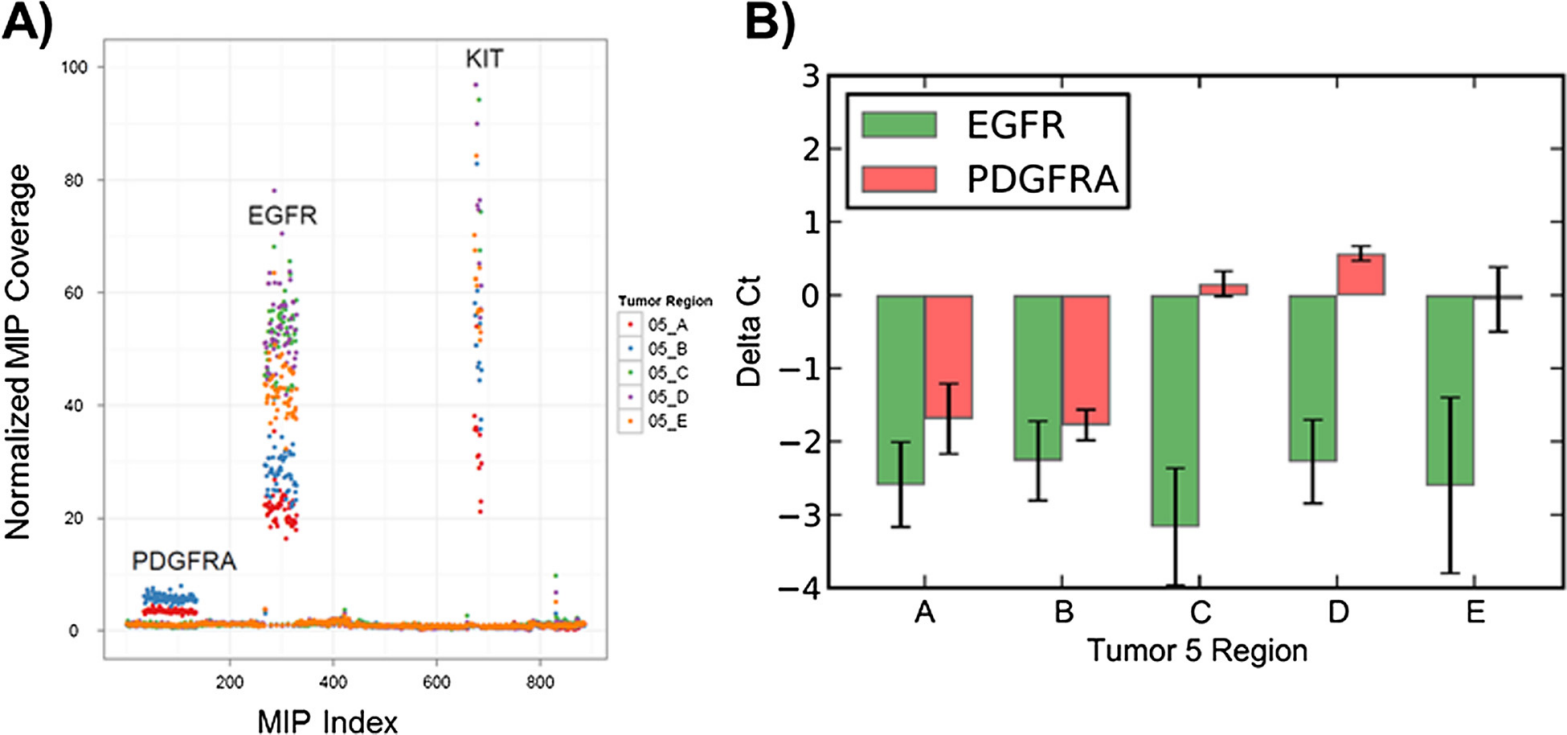
**Heterogeneity of** ***PDGFRA*** **amplification in BI05. (A)** Copy number estimates based on smMIP probe data. *PDGFRA* amplification (labeled) occurs in regions A and B with no amplification in regions C, D or E. **(B)** Results from Taqman qPCR targeting both *PDGFRA* and *EGFR* performed in quadruplicate. *PDGFRA* amplification occurs in regions A and B (between four- and eight-fold amplification) with no significant amplification in regions C, D and E. *EGFR* amplification occurs in all regions of BI05, consistent with MIP sequencing results. Heterogeneity of *PDGFRA* amplification was also confirmed through immunohistochemistry of regions A and E (Figure S9 in Additional file 1). Error bars represent the mean +/- one standard deviation from quadruplicate values.

Our smMIP technique detected amplification of *PDGFRA*, *KIT* and *EGFR* within tumor BI05, an *IDH1*-wild type glioblastoma. In this tumor *EGFR* amplification was seen across all tumor regions, while amplification of both *PDGFRA* and *KIT* was detected in two of five regions (Figure 4A). As *KIT* is located near *PDGFRA* on chromosome 4, shared amplification of these genes is expected [11]. Taqman real-time PCR assays performed in quadruplicate confirmed both the amplification in *EGFR* and the amplification in *PDGFRA* across all assayed regions (Figure 4B). Immunohistochemistry of *PDGFRA* and *EGFR* on tissue adjacent to regions A to E provided additional confirmatory evidence (Figure S9 in Additional file 1).

**Figure 5:**
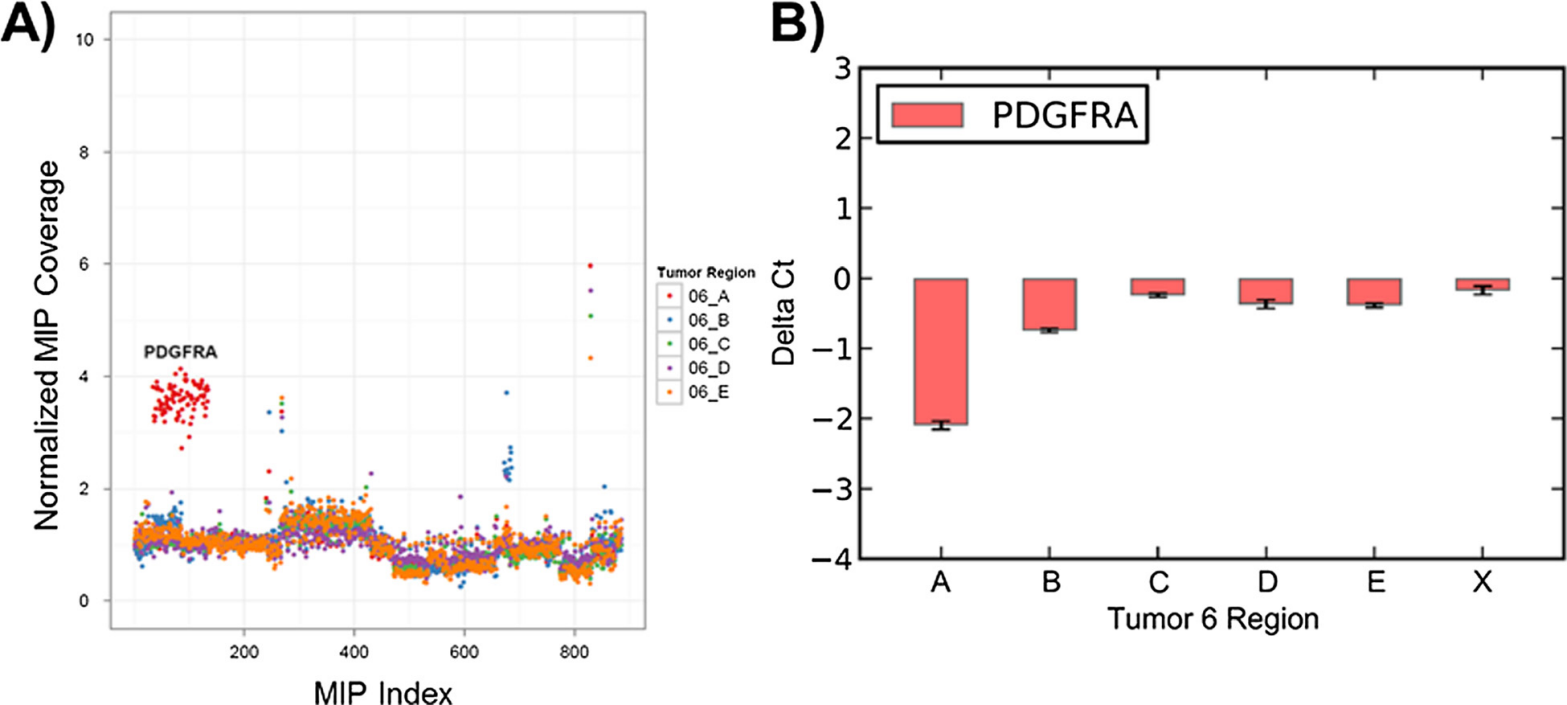
**Heterogeneity of** ***PDGFRA*** **amplification in BI06. (A)** Copy number estimates based on smMIP probe data. *PDGFRA* amplification (labeled) occurs in region A with only mild amplification in region B and no clear detectable amplification in regions C, D or E. **(B)** Results from Taqman qPCR targeting *PDGFRA* performed in quadruplicate. Region X refers to a region of brain tissue grossly uninvolved by tumor. *PDGFRA* amplification occurs in region A (approximately four-fold amplification) with only mild amplification in regions B, C, D and E. Error bars represent the mean +/- one standard deviation from quadruplicate values.

Similarly, we detected heterogeneity of *PDGFRA* amplification within BI06, an *IDH1*-mutant glioblastoma. This tumor had amplification of *PDGFRA* and *KIT* in region A not detected within other regions (Figure 5A). Taqman qPCR confirmed amplification of region A, mild amplification in region B and no amplification in regions C, D and E (Figure 5B). All other regions of this tumor had somatic mutations in *PTEN*, such that reduced tumor cellularity is an unlikely explanation for our observations.

### Additional cases of heterogeneity are potential passenger mutations

A missense mutation in *KRAS* was observed at moderate allele fraction (10% of reads) in region D of the glioblastoma BI12 and was not detected in at least one other region (Table S3 in Additional file 1). As this mutation does not occur within known mutation hotspots and is in a tumor with signs of hypermutation (BI12), the clinical significance of this heterogeneity remains unclear. Other somatic point mutations are heterogeneous across an individual tumor but occur within genes that have another, ubiquitously distributed mutation. BI12 has missense mutations in *PTEN* that are observed in regions A, B and C and not in region D (Table S3 in Additional file 1). This tumor also has another high allele fraction mutation in this gene that is present across all regions of this tumor. A similar scenario is seen in the astrocytoma BI08. Regions D and E of this tumor have low allele fraction point mutations in *TP53*, but all regions share another high allele fraction mutation in the same gene. These results suggest that the heterogeneously observed mutations observed are more likely secondary passenger mutations that occurred after *PTEN* and *TP53* were inactivated in tumors BI12 and BI08, respectively (Table S3 in Additional file 1). An alternative possibility is that tumors may contain distinct subpopulations in which genes were inactivated by a different mutation.

## Discussion

These results demonstrate that intratumoral spatial heterogeneity with respect to clinically relevant genes occurs among multiple types of brain tumors, and spans the mutational spectrum from copy number to point mutations. Across a set of recurrently mutated cancer genes (33 genes examined for point mutations, 21 genes for amplifications), we observed heterogeneity for clinically relevant mutations in 3 of 14 (21%) glial tumors. These include point mutations in *TP53* and *RB1* as well as amplifications in *PDGFRA*/*KIT*. All cases of mutational heterogeneity that we detected in a tumor occur in adjacent regions, consistent with the hypothesis that spatially distinct regions represent divergent subclones of a single tumor.

Historically, in anaplastic oligodendroglioma with intact 1p, mutations in *TP53* were found to stratify outcomes, with median survival of 71 versus 16 months in patients with mutant versus wild-type *TP53*, respectively [12]. While not specifically applying to our patient in whom 1p/19q is deleted, our data demonstrating discrete differences in *TP53* status from different regions within an individual tumor nevertheless show the potential of genetic heterogeneity to confound the assignment of prognostication based on the detection of specific molecular markers.

In addition, decision-making regarding the use of receptor tyrosine kinase inhibitors could be influenced by the status of amplifications/mutations in *PDGFRA*. Our finding of regional heterogeneity of *PDGFRA* within tumors BI05 and BI06 confirms recent work by Sottoriva *et al.* and others and suggests that a single biopsy may not be sufficient to allow for informed application of targeted therapies against these presumed oncogenic drivers [3,4,13]. Clinical decision-making at recurrence will also likely be impacted by regional heterogeneity. Nickel *et al*. [5] compared mutations within a group of 10 genes from 2 regionally distinct samples of a single GBM at initial resection and 2 subsequent recurrences. No heterogeneity was detected at initial resection but heterogeneity of *PIK3CA* and *PTEN* mutation was detected at the first recurrence, and heterogeneity of *PIK3CA*, *TP53* and *EGFR* mutation was detected at the second recurrence.

This study used tumor-adjacent brain to serve as a ‘control tissue’ to help identify somatic mutations. However, after extracting DNA and investigating mutations, we observed that in several cases the grossly normal brain tissue actually contained a large fraction of infiltrating tumor cells. This aspect of our study ultimately complicated the process of calling somatic point mutations (requiring filtering against databases of germline variation) and copy number (necessitating our use of a universal control). While blood would be the most appropriate material to use as a control we did not have institutional review board approval to collect blood at the time these experiments were conducted (subsequently modified to allow for blood collection in the future).

In this study we were able to identify amplification of only a subset of genes of interest, as some genes had too few probes to accurately determine copy number. Our study also did not detect genomic rearrangements and deletions such as the *EGFR* VIII deletion commonly found in GBM. However, one can imagine expanding this assay to consider amplifications and deletions with smMIPs by tiling probes at higher density and incorporating known SNP positions to aid in identifying cases of loss of heterozygosity. One could also capture additional glioma-relevant genes like *IDH1* and *IDH2* by adding probes targeting these genomic regions.

Our investigation focused on regional heterogeneity within a tumor, instead of the microscopic heterogeneity that is likely present within a given tumor biopsy. As we performed the smMIP assay on DNA extracted from tissue pieces that likely contained millions of cells, we would likely miss cases of heterogeneity where only a small population of cells within a biopsy contained a mutation (such as an amplification). Use of techniques such as immunohistochemistry, FISH and, more recently, single cell sequencing remain necessary to characterize the extent of microscopic heterogeneity in tumors.

These results validate the smMIP approach as a scalable and cost-effective platform for deep sequencing of cancer genomes to examine subclonal variation. Despite deeply sequencing multiple sections of 14 tumors, our survey required only one lane of sequencing on the Illumina HiSeq because we focused on well-known gene targets of mutation in cancer. In contrast to the technique used by a similar investigation [5], our method is also easily scaled and amenable to automation with samples processed in 96-well formats. This advantage in scalability enables one to easily assay many more regions (tens to hundreds) per tumor to obtain much finer scale pictures of intratumoral heterogeneity, as we are likely underestimating its extent even here by sampling of only a few regions. While our study represents an improvement over previous studies, analysis of greater number of genes in a greater number of tumors will be necessary to determine rates of regional heterogeneity in different driver mutations across GBMs.

## Conclusions

We find multiple instances of regional heterogeneity in clinically relevant cancer genes within malignant gliomas at the time of diagnosis. We also demonstrate a scalable technique that can be used to efficiently characterize regional genetic heterogeneity for both point mutations and copy number alterations in tumors. Future challenges will include how best to interpret cases of intratumoral heterogeneity and test its impact in the context of clinical trials using targeted therapy approaches.

## Materials and methods

### Samples

Freshly resected brain tumor specimens from adult patients were obtained with informed consent as part of the Genomics Big Idea pilot program (UW/FHCRC). Tissue, patient demographics and final diagnosis were obtained in accordance with protocols approved by the institutional review board at the University of Washington. Tumors were divided into three to five regions, depending on size. Tissue from each region was then subdivided into four pieces for use in next generation sequencing, histology, cell culture and xenotransplantation (Figure 1). In 10 cases, brain grossly uninvolved by tumor was resected to provide adequate surgical access and was utilized as a source of germline or ‘control’ DNA to identify somatic mutations (Table S1 in Additional file 1). These tissues are referred to as regions “X” in this manuscript. For all samples, DNA was isolated from snap-frozen tissue pieces using the QIAGEN DNEasy Blood and Tissue kit (Qiagen, Venlo, Netherlands).

### Targeted capture and sequencing

The smMIP assay was used to genotype candidate genes. Probes were previously designed by Hiatt *et al*. [6] against 33 genes that are commonly mutated in cancer (Table S2 in Additional file 1). Targeted capture and PCR amplification were performed as previously described, except that 200 ng of genomic DNA was used for each sample instead of 500 ng [6]. After smMIP capture, amplified products were pooled and sequenced on a single lane of the Illumina HiSeq 2000 platform with paired 100-nucleotide reads and an 8- nucleotide index read.

### Primary analysis and variant calling

Initial analysis steps through to read mapping were performed as previously described [6], except that instead of constructing a consensus read from tagged smMIP molecules, we chose one read per unique molecular tag event at random for subsequent analysis.

Variants were called using SAMtools, and were filtered for positions with *phred* base quality ≥30, ≥30× coverage and the absence of a neighboring homopolymer run of four bases or more (Table S2 in Additional file 1). To remove common polymorphisms and enrich for likely somatic mutations, we imposed a number of additional requirements, including requiring variants to be observed with an allele balance of at least 5% within a sample, removing variants present within a modified database of the Exome Sequencing Project [14] and 1000 Genomes [15] pilot project that had first been stripped of all COSMIC variants, removing variants that were present at an allele balance of at least 5% in two or more control samples.

### Copy number analysis

We compared read depth of smMIP-targeted regions in each tumor against that of the control tissue BI12 to identify high level gene amplifications in tumors. We restricted the copy number analysis to targets with greater than 30× coverage in control tissue and a GC content ranging from 30 to 60%. To reduce the number of potential artifacts remaining, we removed from consideration (for the purposes of copy number analysis only) 12 genes (*AKT1*, *AKT2*, *CDK4*, *CDKN2A*, *FGFR3*, *HRAS*, *KRAS*, *MYC*, *NRAS*, *SRC*, *STK11*, and *VHL*) that had fewer than 15 probes with sufficient coverage in the control tissue (BI12).

After calculating the ratio of coverage for each probe relative to control tissue from BI12, we used DNACopy [8] to segment genes into discrete levels of coverage and obtain R, the mean ratio of coverage relative to control for each gene. We estimated the copy number for each gene by dividing R for each gene by the median value of R across all genes for each tissue. Genes with ratios above 3 were called as amplified and genes with ratios above 6 were called as highly amplified.

### Sanger validation

DNA from five regions of tumor BI09 were subjected to Sanger sequencing (Genewiz, South Plainfield, New Jersey, USA) against positions within *IDH1*, *TP53* and *RB1*.

### Copy number validation

Tumors with regional heterogeneity in *EGFR* and *PDGFRA* detected using smMIP sequencing were confirmed using Taqman qPCR analysis. DNA from each region was analyzed in quadruplicate using commercially available probes against *PDGFRA* (assay ID: Hs02749151_cn; Life Technologies, Waltham, Massachusetts, USA) and *EGFR* (assay ID: Hs07526740_cn; Life Technologies). Reference primers amplified a fragment from *TERT* (number 4403316; Life Technologies). Finally, to compare sensitivity of the smMIP approach, all regions from all tumors were assayed in duplicate for *EGFR* copy number.

### Immunohistochemistry and FISH

Immunohistochemistry for IDH1 and p53 was performed on 4-micron paraffin sections using mouse anti-human p53 clone (1:2,000 dilution; DAKO, Glostrup, Denmark) and mouse anti-human IDH1 R132H (1:200 dilution; Dianova, Hamburg, Germany). All tumors were investigated for IDH1 mutation by neuropathology, while only a subset of tumors was investigated for p53 expression by immunohistochemistry (Table S1 in Additional file 1). Immunohistochemistry for EGFR and PDGFRA was performed on 5- to 6-micron paraffin sections using mouse anti-human EGFR, clone 2-18C9 (pharmDx kit, DAKO) and rabbit anti-human PDGFRα, clone D1E1E (1:500 dilution; Cell Signaling, Danvers, Massachusetts, USA). Dual-color *EGFR* FISH was performed using commercially available probes (LSI *EGFR* SpectrumOrange/CEP 7 SpectrumGreen, number 32-191053; Abbott Molecular, Chicago, Illinois, USA) with DAPI counterstain using standard methods. Slides were imaged using an Olympus DP72 digital camera mounted on a Nikon E400 microscope. Fifty nuclei were scored for each region. *EGFR* amplification was called if more than 10% of nuclei either contained many *EGFR* signals or exhibited a *EGFR:CEP7* ratio greater than 2. 1p19q deletion FISH was performed using commercially available probes (number 04 N60-020, Abbot Molecular) using standard methods.

### Data availability

All sequence data from smMIP capture experiments have been deposited in the NCBI Sequence Read Archive (SRA) under accession number SRP049298.

## Additional file

Additional file 1:
**Supplementary figures, tables and their associated legends.**

## Abbreviations

FISH: fluorescence *in situ* hybridization
GBM: glioblastoma multiforme
MIP: molecular inversion probe
PCR: polymerase chain reaction
qPCR: quantitative PCR
smMIP: single molecule molecular inversion probe
