# Supplementary material for: Deep sequencing of multiple regions of glial tumors reveals spatial heterogeneity for mutations in clinically relevant genes
Source: Genome Biol. 2014 Dec 3;15(12):530. doi: 10.1186/s13059-014-0530-z (PMC4272528; doi:10.1186/s13059-014-0530-z)
Supplement: Additional file 1: — Supplementary figures, tables and their associated legends. [file 13059_2014_530_MOESM1_ESM.pdf]

Supplementary Material

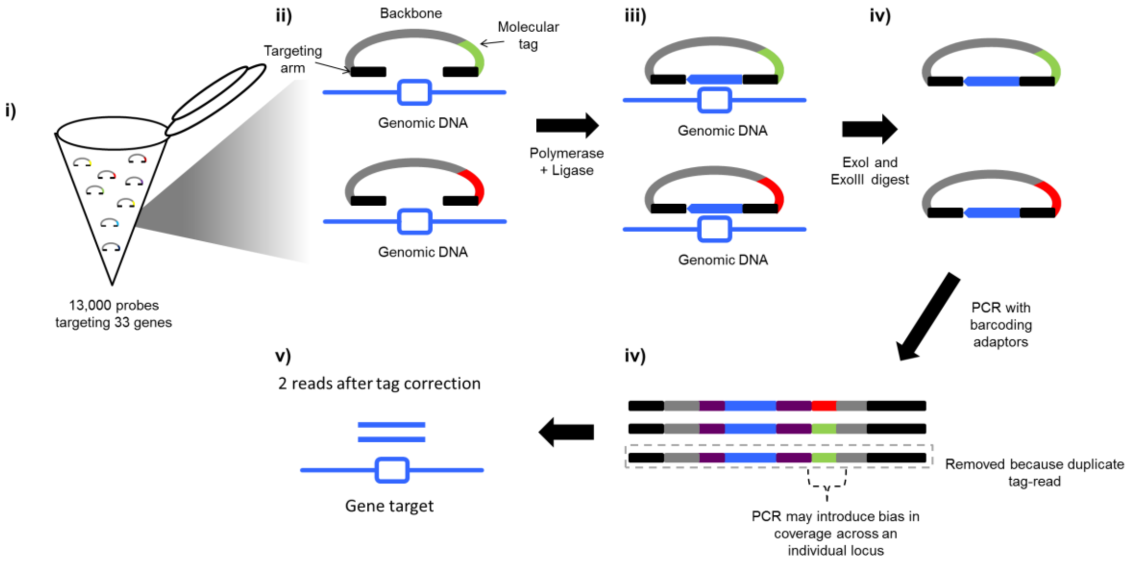

Supp. Figure 1: Overview of Molecular Inversion Probe Strategy (detailed). i) genomic DNA from each region is added to a tube containing a mixture of probes targeting the exons of 33 genes. For clarity, we focus on one targeted region here. ii) A single molecule Molecular Inversion Probe (smMIP) consists of two regions complementary to a target of interest, a common backbone sequence and a 12bp molecular tag (used in error-correction). iii) After a polymerase gap-fill and ligation, each target sequence is captured to create a circular molecule of DNA. iv) Exonuclease (ExoI + ExoIII) digest removes remaining genomic DNA template and single stranded smMIP probes. v) After inverse PCR (with barcoding adaptors) against the common backbone, some targets are nonuniformly amplified. These instances are removed after molecular tag-correction. Barcode sequences (not shown) allow capture products from multiple individual tumors or regions to be pooled on a single sequencing lane.

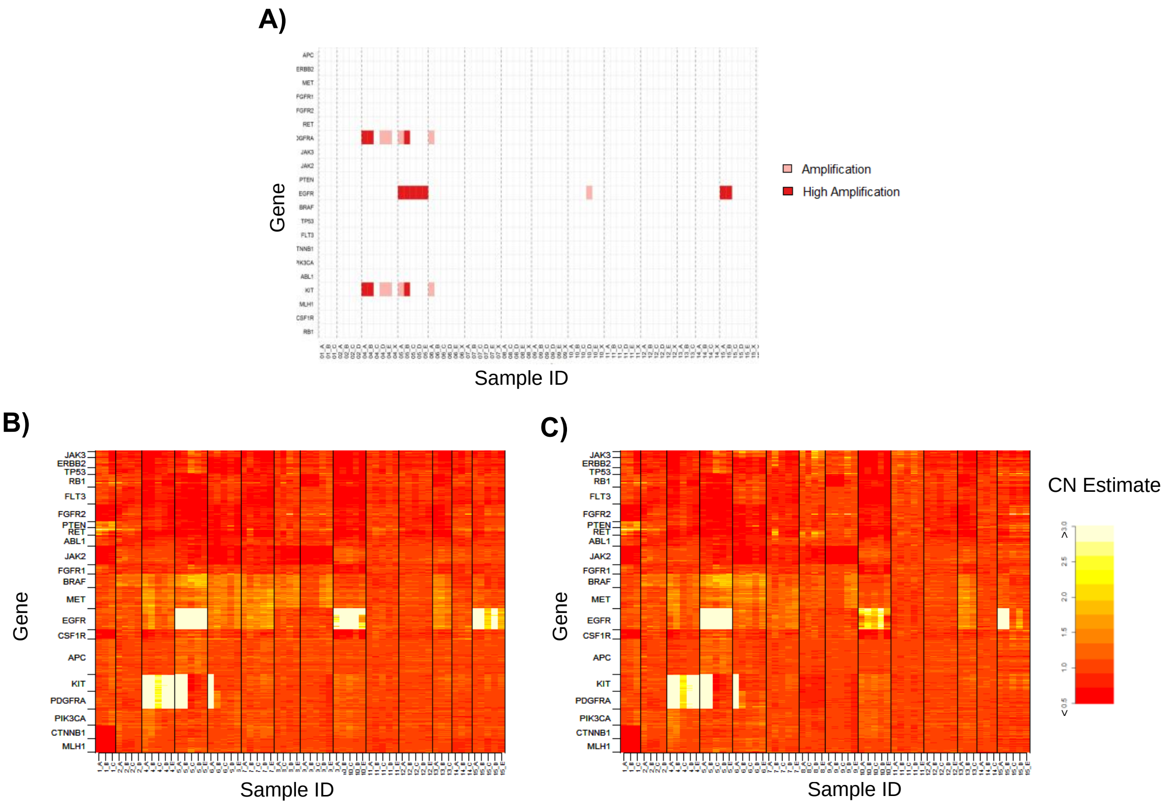

Supp. Figure 2: a) Copy number calls from MIP captures using matched control tissue for samples BI04, BI06, BI07, BI08, BI09, BI10, BI11, BI12, BI14 and BI15, and a universal control (BI12) for samples BI01, BI02, BI05 and BI13 (as for the latter, matched control tissue was not available). Amplification indicates genes with coverage three-fold higher than median coverage across a sample. High Amplification indicates genes with coverage six-fold higher than median coverage across a sample. Notably, this analysis does not detect EGFR amplification in regions A, B and C of BI10 and region D of tumor BI15, events that were detected with the use of the universal control (BI12; see **Figure 2** of main text). These EGFR amplifications were also detected using Taqman qPCR (**Supp. Figure 3**). Careful review indicates the reason for discrepancy is likely due to increased tumor contamination within the respective matched control tissues (region "X" in tumors **BI04, BI07, BI08** and **BI10** in **Supp. Table 3**). In those cases, the control tissue exhibited a high allele fraction of mutation in known cancer genes. For this reason, we chose to rely on the results of analysis where all tumors were matched with BI12 as a universal control. Panels **b)** and **c)** show the raw data used to call copy number using universal and matched controls, respectively. Tumor regions are shown on the x-axis with vertical lines separating regions from different tumors. Probes (grouped by gene) are shown on the y-axis. The color represents the read depth at each probe normalized against the median read depth across all other probes from the same tumor sample. Use of a "universal control" enables better detection of high-level EGFR amplifications in multiple regions of both tumor BI10 and BI15. We used high thresholds to call a gene as amplified (CN estimate>3). Blocks of higher signal may correspond to aneuploidy; however thresholds were not set for this sensitivity.

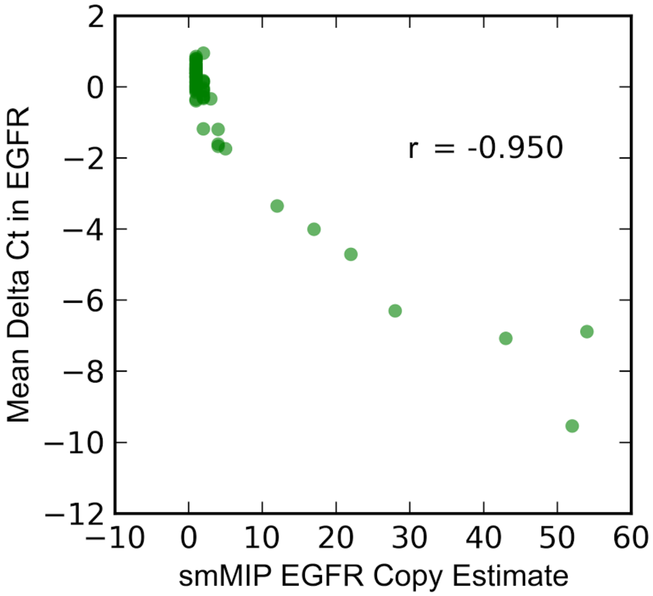

Supp. Figure 3: Validation of EGFR gene estimates Correlation with of copy number estimates from smMIP vs. Taqman for EGFR**.** Taqman experiments were performed in duplicate for EGFR across all 62 regions investigated in this study. smMIP and Taqman copy number estimate were highly correlated with an R^2 of .90. Importantly, all high-level amplifications of EGFR (delta Ct ≤ -2) were identified by the smMIP assay.

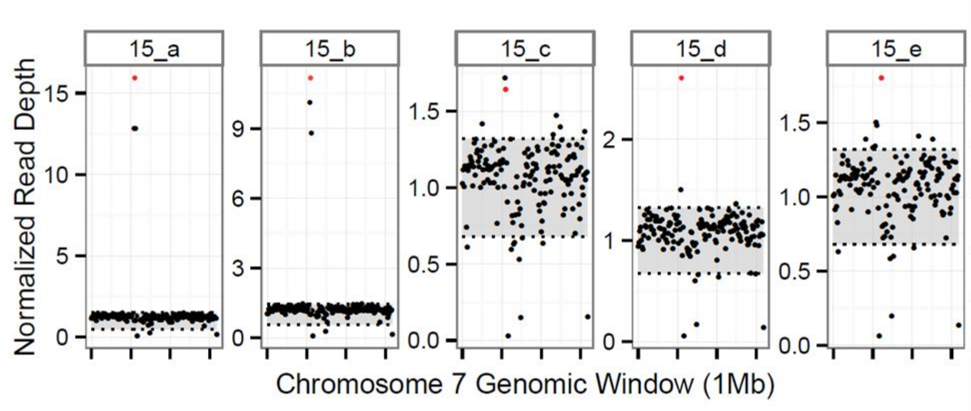

Tumor	MIP_CN	WG_CN
15_a	17	15.9
15_b	12	11.2
15_c	2	1.6
15_d	3	2.6
15_e	2	1.5

Supp. Figure 4: Validation of EGFR copy number by low-pass whole genome sequencing. DNA isolated from regions A-E in BI15 were subjected to light genome sequencing on the Illumina Miseq. Read depth within 1 Mb intervals across Chromosome 7 is normalized with respect to mean read depth across all chromosomes within each sample (see **Supplementary Methods**). Normalized read depth from whole genome sequencing within the 1 Mb region containing *EGFR* is highlighted in red within CN plots. Copy number of *EGFR* (WG_CN in table) from low-pass whole genome sequencing was compared with those estimates obtained using the MIP assay (MIP_CN). Regions A and B contain high-level amplification in the region containing EGFR while a similar amplification is not seen within regions C, D and E.

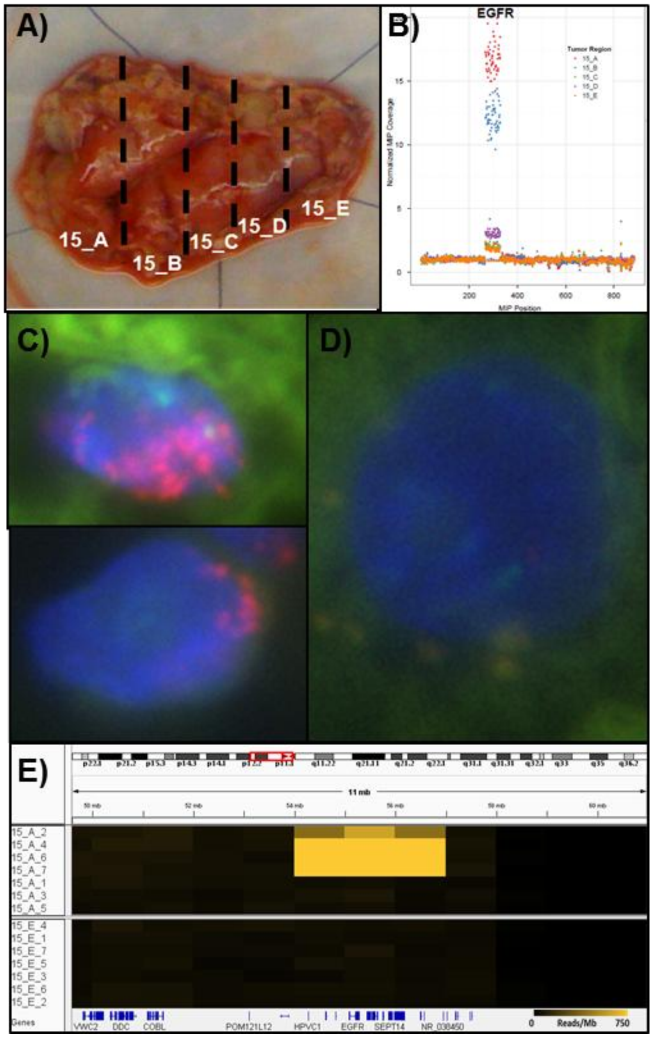

Supp. Figure 5: Measured *EGFR* amplification heterogeneity a result of varying levels of stromal contamination in BI15. a) GBM tumor used in dissection. **b)** Copy number estimates based on smMIP probe data. *EGFR* amplification (labeled) was called in regions A and B with only mild amplification detected in region C, D and E. Histologic examination and whole genome sequencing (**Supp. Figure 6**) suggested a marked decrease in tumor cellularity in regions C, D and E which likely accounted for the difference in copy number. **c)** and **d)** show representative FISH detection of *EGFR* amplification in region A (left images) and its absence in region E, respectively. Unprocessed images were obtained using a dual pass filter for spectrum orange and spectrum green and spectrum blue (DAPI). **e)** Validation of *EGFR* amplification in region A using single cell sequencing. Single cells from regions A and E were flow sorted, amplified and sequenced on the Illumina Miseq, resulting in 100,000 reads per sample. Copy number profiles were created by plotting read depth across the genome in 1 Mb intervals, with color of each genomic region corresponding to the number of mapping reads per interval. Four of seven cells from region A (15_A_2, 15_A_4, 15_A_6 and 15_A_7) have high level *EGFR* amplification while zero of seven cells in region E have similar amplification.

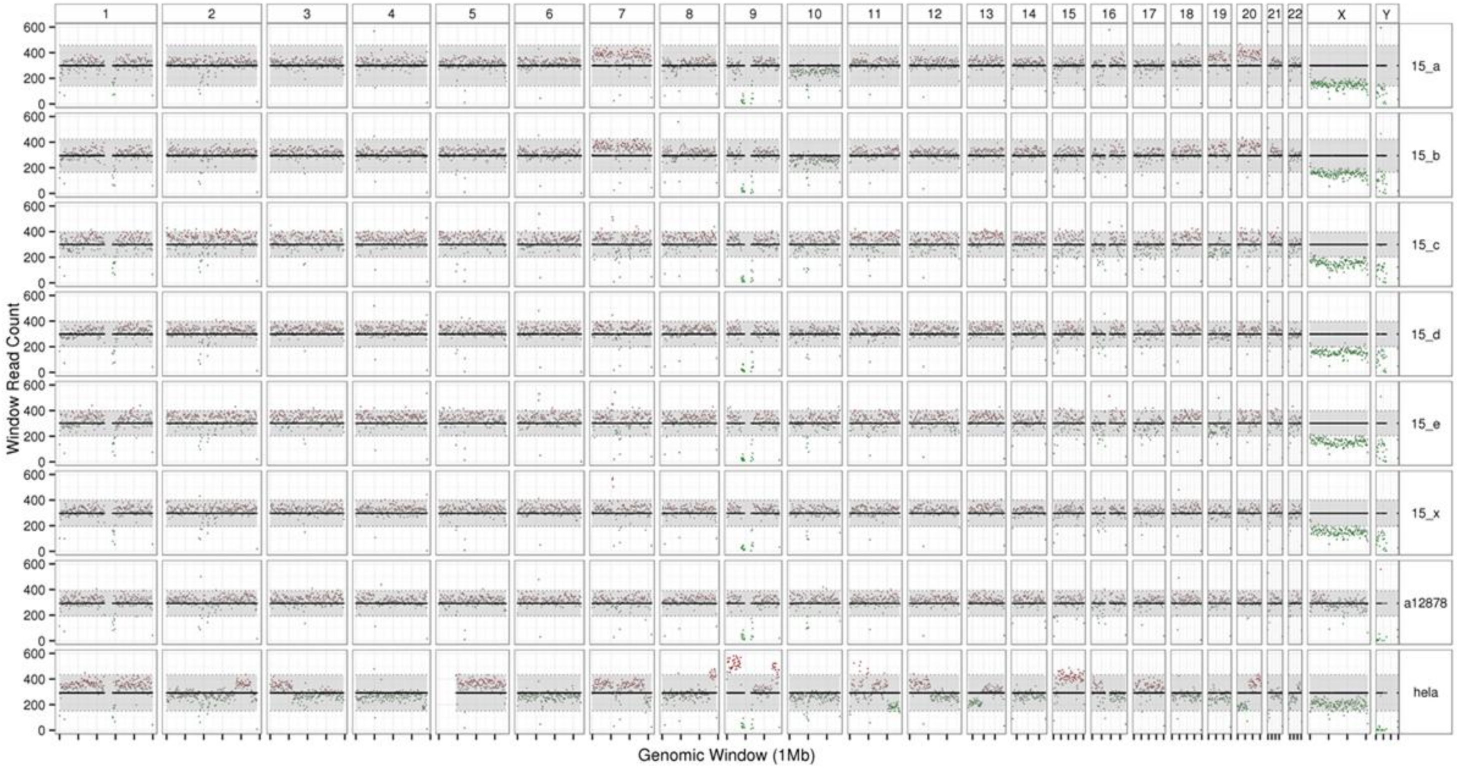

Supp Figure 6: Whole genome copy number profiles of regions A-E in BI15. To identify other possible copy number alterations that may be shared across all tumor sections, DNA isolated from regions A-E from BI15 (shown as 15_a - 15_e), the corresponding control region X (15_x) and two unrelated cell lines (NA12878 and HeLa) were subjected to light genome sequencing on the Illumina Miseq. 500,000 reads per sample were aligned to the hg19 reference and copy number is shown across the genome in 1 Mb intervals. Regions A and B of BI15 share gain in chromosome 7 loss of chromosome 10. However, no gross chromosomal aberration was shared across all tumor regions. Black line corresponds to the mean coverage across all 1 Mb windows in autosomes. Shaded regions correspond to the region 1 S.D. below and above mean coverage for each sample. Two cell lines (12878 and HeLa) derived from female individuals are shown for comparison. Chromosome X appears as lost in all regions from tumor BI15 (including control) of the tumor as it was derived from a male patient.

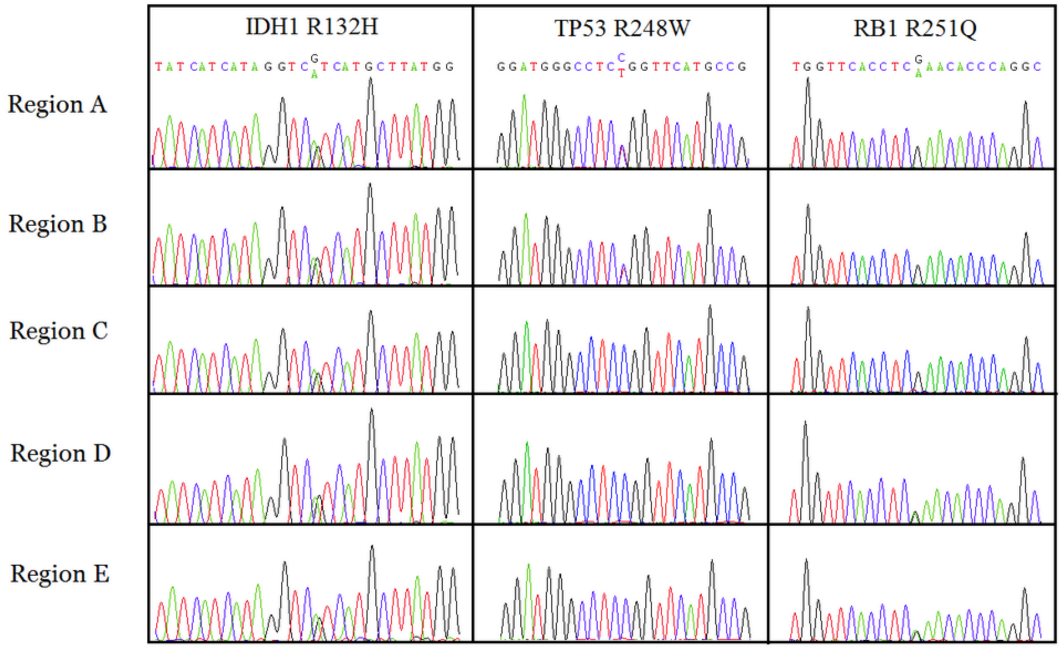

Supp. Figure 7: Sanger validation of *TP53* and *RB1* heterogeneity in tumor BI09. We performed Sanger sequencing across three different loci from 5 regions of tumor BI09. All five regions (A-E) share mutations in *IDH1*. Tumor regions A and B have detectable mutations in *TP53*, while regions D and E have detectable mutations in *RB1*.

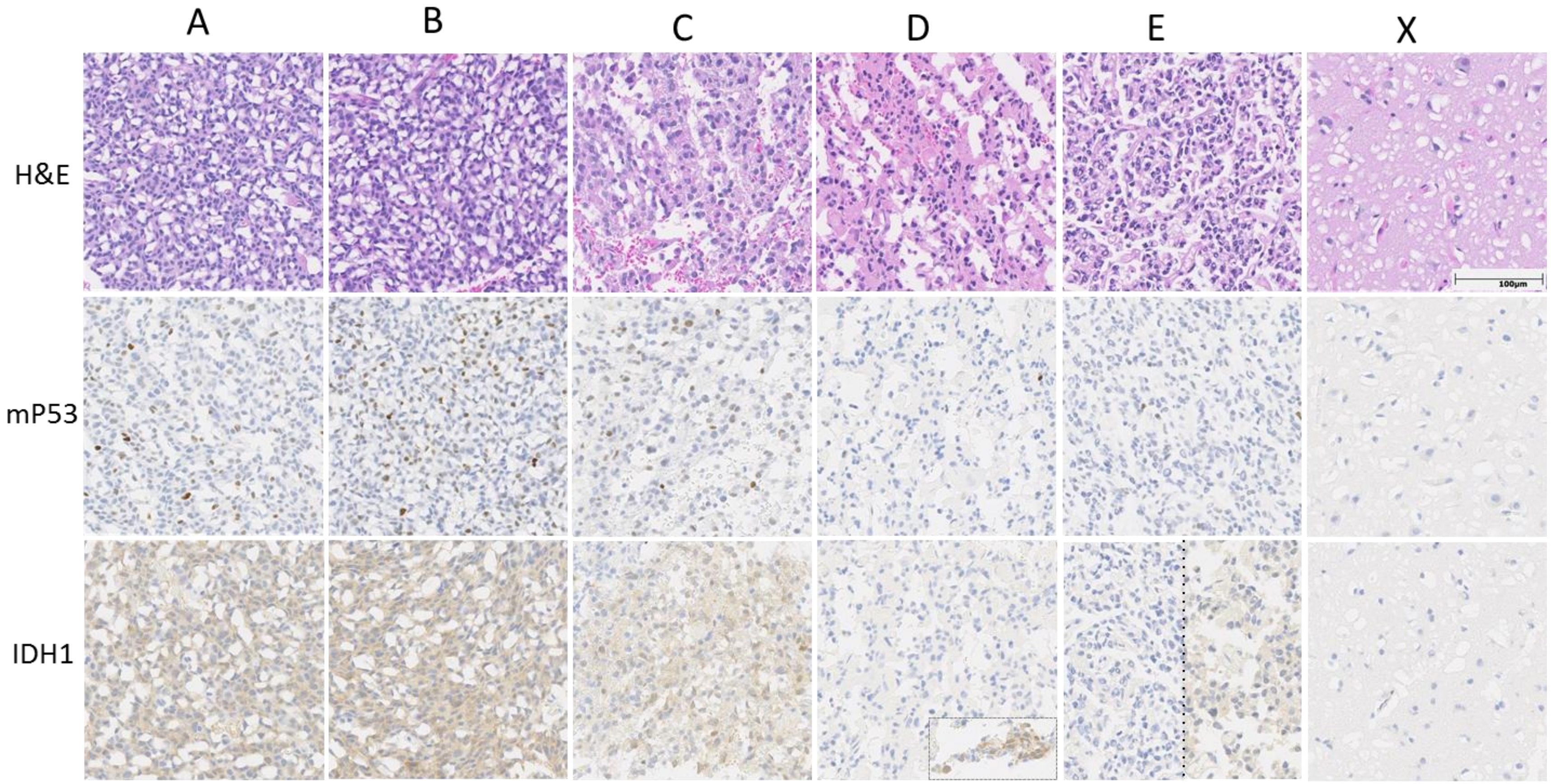

Supp. Figure 8: H&E and immunohistochemical (IHC) staining of p53 and IDH1 in tumor BI09. IHC staining of p53 and IDH1 is shown across tumor regions A-E and corresponding control region X from tumor BI09. The pattern of staining differs across each of the five regions (A-E) and is consistent with the intratumoral heterogeneity identified with by sequencing. Partitioning of IDH1 photographs D and E illustrates that IDH1 heterogeneity was also present within these sections.

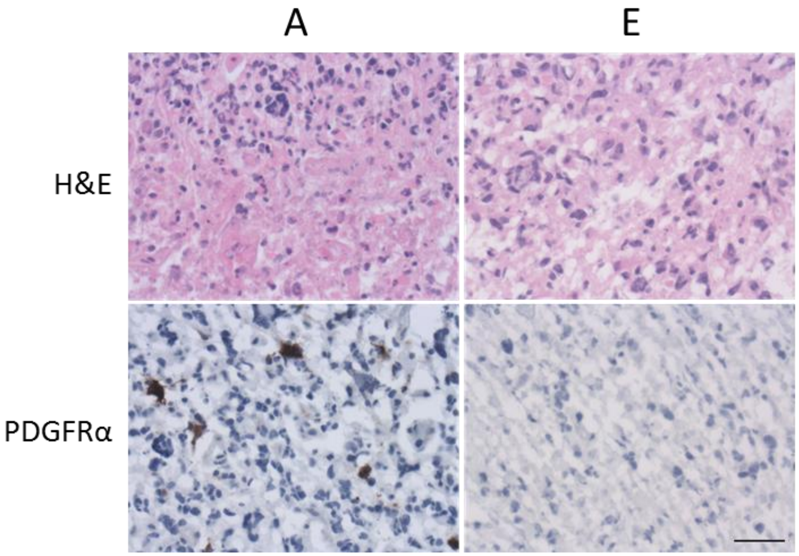

Supp. Figure 9: H&E and PDGFRα IHC staining of regions A and E in tumor BI05. IHC of regions A and E reveals differential staining of PDGFRα, with staining prominent in region A and not in region E. This is consistent with genomic findings: with amplification of the PDGFRA gene observed in regions A and B but not C, D or E. Original magnification 40x. Scale bar indicates 30 microns. EGFR IHC revealed robust expression across all regions (not shown).

Sample Name	Tumor Type*	Grade*	Age at Surgery	IDH1 status	p53 status	1p19q status	Control tissue	# Samples (Mutation)	# samples (CN)
BI_01	Ependymoma	III	33	mutant	NA	NA	N	3: A,B,C	3: A,B,C
BI_02	GBM	IV	29	mutant	mutant	NA	N	4: A,B,C,D	4: A,B,C,D
BI_04	GBM	IV	67	wt	NA	NA	Y	5: A,B,C,D,E	5: A,B,C,D,E
BI_05	GBM	IV	60	wt	NA	NA	N	5: A,B,C,D,E	5: A,B,C,D,E
BI_06	GBM	IV	63	mutant	NA	NA	Y	5: A,B,C,D,E	5: A,B,C,D,E
BI_07	GBM	IV	62	mutant	NA	NA	Y	5: A,B,C,D,E	5: A,B,C,D,E
BI_08	Astrocytoma	III	35	mutant	mutant	Not-del	Y	4: A,C,D,E	4: A,C,D,E
BI_09	AO	III	72	mutant	mutant	Co-del	Y	5: A,B,C,D,E	5: A,B,C,D,E
BI_10	GBM	IV	73	wt	NA	NA	Y	5: A,B,C,D,E	5: A,B,C,D,E
BI_11	GBM	IV	41	wt	NA	NA	Y	5: A,B,C,D,E	5: A,B,C,D,E
BI_12	GBM	IV	63	wt	NA	NA	Y	5: A,B,C,D,E	5: A,B,C,D,E
BI_13	GBM	IV	68	wt	NA	NA	N	3: A,B,C	3: A,B,C
BI_14	Astrocytoma	II	19	mutant	NA	NA	Y	3: A,B,C	3: A,B,C
BI_15	GBM	IV	57	wt	NA	NA	Y	5: A,B,C,D,E	5: A,B,C,D,E

Supp. Table 1: Tumors investigated in this study. Our sample set includes a total of 62 spatial sections from 14 glial tumors. All tumors were grade III or higher, with one exception. Clinical information regarding IDH1, p53 and 1p19q mutation as determined by pathology is shown. For a total of 10 tumors matched “Control tissue” was available in the form of adjacent brain tissue that appeared to be grossly uninvolved with tumor. IDH1 status was measured by IHC against IDH-R132H mutant. GBM, Glioblastoma Multiforme; wt, wild-type; CN, copy number; AO: Anaplastic Oligodendroglioma; NA: Not Available;
*Diagnosis based on neuropathology report, which is based on the highest degree tumor available at the time

Gene	# of Targeted Coding Bases	Median % bases >30x coverage
ABL1	3613	96.5%
AKT1	1501	84.5%
AKT2	1561	96.7%
APC	8828	96.4%
BRAF	2389	92.3%
CDK4	947	98.3%
CDKN2A	1293	73.9%
CSF1R	3082	98.0%
CTNNB1	2386	97.6%
EGFR	4288	97.8%
ERBB2	3832	89.0%
FGFR1	2776	98.1%
FGFR2	2915	98.0%
FGFR3	2679	87.9%
FLT3	3075	95.5%
HRAS	722	97.2%
JAK2	3511	97.8%
JAK3	3421	92.9%
KIT	3042	98.3%
KRAS	739	99.1%
MET	4285	98.9%
MLH1	1963	97.9%
MYC	1403	97.6%
NRAS	595	98.8%
PDGFRA	3356	98.8%
PIK3CA	3267	96.5%
PTEN	1257	88.8%
RB1	2838	94.5%
RET	3599	95.4%
SRC	1666	91.7%
STK11	1377	89.9%
TP53	1448	94.6%
VHL	510	94.9%

Supp. Table 2: Genes Targeted by single molecule Molecular Inversion Probe (smMIP) assay with capture efficiency. Our probes target the coding sequence of 33 cancer related genes. The number of coding bases targeted and capture efficiency is shown across these genes here. Median % bases >30x coverage represents the median percent of targeted coding bases that have greater than 30x coverage across all samples captured. Positions were required to have greater than 30x coverage in order to be considered for mutation calling.

					Allele Balance within each region						Coverage (tag-corrected)					
**Tumor**	**Chr**	**Position**	**Gene**	**Mutation type**	**X**	**A**	**B**	**C**	**D**	**E**	**X**	**A**	**B**	**C**	**D**	**E**
BI01	4	55593464	*KIT*	missense	NA	0.5093	0.5064	0.5039	NA	NA	NA	699	1637	772	NA	NA
BI01	17	7577121	*TP53*	missense	NA	0.9442	0.9688	0.9606	NA	NA	NA	1325	2661	635	NA	NA
BI02	4	55593464	*KIT*	missense	NA	0.5181	0.5226	0.5074	0.544	NA	NA	1355	597	808	897	NA
BI02	17	7577124	*TP53*	missense	NA	0.8952	0.8488	0.8918	0.9006	NA	NA	1680	820	915	1147	NA
BI04	17	7577535	*TP53*	missense	0.1792	0.7706	0.642	0.2302	0.5122	0.7401	1557	1308	1014	1690	1478	1443
BI05	13	28626716	*FLT3*	missense	NA	0.709	0.7306	0.9126	0.9792	0.8601	NA	409	657	206	192	243
BI05	17	37866342	*ERBB2*	missense	NA	0.1538	0.0805	0.0345	0	0.1481	NA	65	87	58	33	54
BI06	10	89711874	*PTEN*	splice	0	0.5143	0.7172	0.5348	0.3033	0.6885	125	175	396	230	244	183
BI07	10	89624273	*PTEN*	missense	0.1956	0.5303	0.3749	0.5211	0.4	0.6341	675	413	939	545	215	246
BI08	17	7577120	*TP53*	missense	0.4149	0.7721	NA	0.6099	0.73	0.814	1157	1009	NA	1033	3834	645
BI08	17	7578204	*TP53*	missense	0.0009	0	NA	0	0.1362	0.0747	1090	787	NA	867	3341	629
BI09	13	48936984	*RB1*	missense	0	0	0	0	0.4451	0.4333	763	369	363	387	1813	630
BI09	17	7577538	*TP53*	missense	0.0069	0.4975	0.4342	0.0045	0.0005	0.0007	1732	1598	1428	1539	4208	1507
BI10	17	7577022	*TP53*	stop-gained	0.0611	0.8765	0.8404	0.6253	0.645	0.9228	1440	502	1247	1073	1690	557
BI12	3	41266113	*CTNNB1*	missense	0	0.3684	0.3737	0.4512	0.4455	0.3493	568	1056	1132	1106	918	355
BI12	3	178921435	*PIK3CA*	missense	0	0.2032	0.142	0.2953	0.1928	0.2808	721	1629	1676	1497	1385	616
BI12	5	112102891	*APC*	missense	0	0.3954	0.3882	0.4678	0.4406	0.3416	448	951	930	902	799	363
BI12	5	112177421	*APC*	missense	0.0023	0.374	0.3708	0.4224	0.4614	0.3527	1318	3032	2964	2919	2484	1069
BI12	7	116340036	*MET*	missense	0	0.0726	0.0212	0.0637	0.1243	0.0226	629	1294	1322	1302	1038	443
BI12	9	5055669	*JAK2*	missense	0	0.3764	0.3005	0.3973	0.4524	0.366	858	2115	2020	1787	1786	817
BI12	10	89685290	*PTEN*	missense	0	0.0009	0.106	0.0363	0.001	0	1401	2124	2255	2206	1917	886
BI12	10	89692790	*PTEN*	missense	0	0.5278	0.4972	0.6395	0.5754	0.5084	668	1061	1090	1057	862	419
BI12	10	89711910	*PTEN*	stop-gained	0	0	0	0.0056	0.1242	0	320	446	755	536	451	182
BI12	12	25380240	*KRAS*	missense	0.0011	0	0.0026	0.0062	0.0974	0.0013	887	2119	1919	1940	1684	766
BI12	13	28588626	*FLT3*	missense	0.0028	0.3483	0.3383	0.6072	0.4048	0.2731	358	669	677	499	583	260
BI12	13	28610078	*FLT3*	missense	0	0.3484	0.3467	0.5534	0.4138	0.3245	327	620	672	506	580	265
BI12	13	28636062	*FLT3*	missense	0	0.4107	0.4206	0.6316	0.5	0.3111	63	112	126	114	90	45
BI12	13	49039143	*RB1*	missense	0	0.3867	0.3962	0.2616	0.4737	0.3333	87	150	212	172	152	66
BI12	17	7578199	*TP53*	missense	0	0.3466	0.3272	0.3974	0.4112	0.3009	452	929	865	780	766	319
BI12	17	7579355	*TP53*	missense	0	0.403	0.3103	0.4209	0.4333	0.2818	188	263	290	297	270	110
BI14	5	149449827	*CSF1R*	missense	0.4326	0.3538	0.4058	0.3844	NA	NA	675	277	855	588	NA	NA
BI14	17	7578263	*TP53*	stop-gained	0.0487	0.012	0.4054	0.3719	NA	NA	719	249	782	691	NA	NA
BI15	17	37866422	*ERBB2*	missense	0.506	0.5057	0.5036	0.4346	0.5371	0.484	747	350	417	260	391	281

Supp. Table 3: Protein-altering candidate somatic mutations. Allele balance of protein-altering candidate somatic mutations across all tumor regions are shown. Tumors were divided into multiple regions (A-E) with a corresponding “control tissue” (X) available from 10 tumors. Candidate mutations were not previously observed in a database derived from >5,000 exomes from the Exome Sequencing Project (ESP) that had been modified to remove positions also found in COSMIC. In several cases (e.g. BI04, BI07, BI08 and BI10) the “control tissue” (X) exhibited a high allele fraction of mutation in known cancer genes (e.g. TP53). We concluded that this was the result of contamination of tumor cells within control tissue and subsequently used a “universal control” for calling copy number alterations (BI12). For several tumors, we did not sequence all five regions plus a control tissue. For these tumors, relevant regions are marked as “NA” or not available.

**Sample Name**	**Tumor Type**	**Grade**	**p53**	***RB1***	***IDH1***
Clinical Pathology Results*	AO	III	NA	NA	mutant (IHC)
09_A	AO	III	mutant	wt	mutant (Sanger)
09_B	AO	III	mutant	wt	mutant (Sanger)
09_C	OA	II	wt	wt	mutant (Sanger)
09_D	OA	II	wt	mutant	mutant (Sanger)
09_E	OA	II	wt	mutant	mutant (Sanger)

Supp. Table 4: Histologic grade correlates with TP53 mutation status in BI09. A pathologist scored sections from each of five regions A-E of BI09 to determine each histopathological type and grade. Results are shown here along with results from DNA sequencing. AO: Anaplastic Oligodendroglioma; OA: Oligoastrocytoma; NA: Not Applicable. wt, wild-type. IDH1 status was measured either by IHC against IDH-R132H mutant (IHC), or by Sanger sequencing (Sanger). *Diagnosis based on neuropathology report, which is based on the highest degree tumor available at the time

Supplementary methods:

Single cell sequencing

Single nuclei copy number analysis was performed as previously described in Baslan et al. 2012 (PMID: 22555242) with two modifications. Briefly, individual nuclei were either isolated from tumor regions A and E from BI15 by mincing tumor tissue in nuclei lysis buffer or isolated from a HapMap cell line GM12878 (Coriell) directly. Suspended nuclei were passed through a 0.2 um filter and sorted on an FACS Aria cell sorter. Sorted cells were placed into individual tubes, amplified using the PicoPLEX (Rubicon Genomics) single cell amplification kit and prepared for sequencing using the Nextera library preparation kit (Illumina). Libraries were sequenced on the Illumina Miseq using paired-end 100 bp reads. 100,000 reads from each single cell library were mapped to the human hg19 reference. Genomic copy number profiles were created by plotting the number of reads mapping across 1 Mb intervals across the reference genome. While the number of reads made identification of smaller amplifications/deletions difficult, cells with EGFR amplification also appeared to have a deletion of chromosome 10.

Whole genome sequencing

Light whole genome sequencing was performed on DNA isolated from multiple regions of BI15 as well as DNA extracted from the Coriell cell line 12878. Purified DNA was fragmented by sonication with the Covaris S2 instrument. Shotgun sequencing libraries were prepared using the KAPA library preparation kit (Kapa Biosystems) with sample barcoding following manufacturer's instructions. All libraries were sequenced on Miseq instruments (Illumina) using paired-end 100-bp reads. Copy number profiles were generated as described in "Single Cell Sequencing".
